# A hierarchical dataset on multiple energy consumption and PV generation with emissions and weather information

**DOI:** 10.1038/s41597-025-06010-8

**Published:** 2025-10-31

**Authors:** Hanjiang Dong, Jizhong Zhu, Chi-yung Chung, Zipeng Liang, Haosen Yang, Xiyu Wen

**Affiliations:** 1 School of Electric Power Engineering, South China University of Technology, Guangzhou, 510641 Guangdong Province China; 2 Department of Electrical and Electronic Engineering, The Hong Kong Polytechnic University, Hong Kong SAR, China

**Keywords:** Energy supply and demand, Energy management, Energy conservation, Energy and behaviour

## Abstract

This study constructs a multi-source and hierarchical dataset of energy consumption, photovoltaic (PV) power generation, greenhouse gas (GHG) emissions, and weather information, dubbed Hierarchical Energy, Emissions, and Weather (HEEW). This dataset contains 11,987,328 records for 147 individual buildings, four aggregated communities, and the entire region, which is structured as time-series tables indexed by building ID and timestamps from 1 January 2014 to 31 December 2022. It includes 13 hourly variables as follows. Energy records involve PV output and total energy consumption of electricity, heat, and cooling loads. Weather involves temperature, dew point, humidity, wind speed, wind gust, pressure, and precipitation. GHG emissions are estimated as the net values between the emissions from energy consumption and the offset by PV output. To ensure the feasibility, we develop a two-stage baseline data cleaning scheme, available at GitHub, where missing values are imputed, and abnormal values are corrected as artificial ground truth. The real-world dataset at figshare in CSV format serves as benchmarks for imputation, anomaly detection, clustering, decomposition, classification, forecasting, and optimization.

## Background & Summary

Utilities such as power systems, information internet infrastructures, transportation networks, natural gas pipelines, and district heating systems are generally operated in an autonomous manner. The concepts of Energy Internet and Integrated Energy Systems, which facilitate the coordination of myriad systems or networks, represent promising approaches for penetrating renewable power generation and promoting net-zero greenhouse gas (GHG) emissions^1^. Through the increasing deployment of smart meters and sensors, practitioners collect more refined observations to offer better energy supply services. For instance, customized services are available for residential, industrial, commercial, and other types of consumers; flexible loads, such as heating, ventilation, and air conditioning, can actively participate in integrated demand responses for penetrating renewables^2,3^. Machine learning, deep learning, and data-driven optimization approaches require data sources to estimate the states of energy systems^4,5^, predict their future conditions^6–8^, and optimize energy generation, distribution, and utilization^2,3,9^. Hence, it is imperative to underscore the criticality of data sources to energy system management.

Due to privacy and information security, data sources are not always accessible, especially in a deregulated market. Presently, complete datasets on energy systems mainly involve electricity consumption^10–12^, renewable energy generation^10,11^, and meteorological information (widely accepted as influential factors for energy system operations)^10–12^, structured in a multi-source manner^10,12^ and hierarchical fashion^12^. Multi-source datasets that involve multiple loads, photovoltaic (PV) panels, or other components are desirable for managing decentralized energy systems such as microgrids. Hierarchical datasets contribute to the interactions between microgrids and centralized energy systems such as power distribution systems. However, the lack of heat and cooling energy consumption may make these datasets unsuitable for analyzing the interactions among multiple energy sectors.

In the context of energy systems penetrated with renewables, many studies prefer a sufficient, complete dataset of multiple energy consumption, renewable power generation, GHG emissions, and weather information. In practice, the number of suitable multiple energy datasets is relatively limited compared to those targeted at power systems, which may constrain further investigations^1^. Generally, only a few types of multiple energy consumption of individuals are provided in the multiple energy dataset, in which the record of renewable power generation is not considered, thus limiting the implementation of the dataset. For instance, the electricity and thermal energy consumption of 38 houses in Germany is contained in the dataset without other data sources^13^. The reality is that multiple energy datasets incorporating multi-source information are significantly less common than those focusing solely on electricity. Furthermore, the record of GHG emissions has not yet been included in current multiple energy datasets. On the other hand, the length of multiple energy datasets is often within a year or several months. It is far from sufficient for leveraging the power of deep learning methods compared with electricity-only datasets^1,6^. There is still a conspicuous absence of multi-source energy datasets with a hierarchical structure (see Fig. 2), from individuals to aggregations and up to the top level, despite the fact that many raw data sources remain messy and insufficiently prepared for reproducibility, customization, and further development.

Hence, this study develops a sufficient multi-source and hierarchical dataset by integrating diverse data sources of multiple energy consumption (e.g., electricity, heat energy, and cooling energy), PV power generation, GHG emissions, and weather information (temperature, dew point, humidity, wind speed, pressure, and precipitation), dubbed Hierarchical Energy, Emissions, and Weather (HEEW). The dataset ranges from 1 January, 2014, to 31 December, 2022, providing information on 147 individual buildings in the entire region. The multi-source data records originating from these buildings can be aggregated to form the combined datasets of the four communities. Then, the aggregated records from these communities can be synthesized to produce a hierarchical dataset representative of the entire region. The dataset can be used for investigations on imputation, anomaly detection, clustering, decomposition, classification, forecasting, and optimization. For reproductivity, customization, and development, we believe this real-world dataset can serve as the benchmark of time series sequence or tabular data to improve the management of multiple energy systems and decentralized systems.

In HEEW, the data source of multiple energy consumption has been widely used in energy demand forecasting, energy trade optimization, energy system simulation, system sustainability assessment, and other topics. Specifically, existing studies identify the complicated, influential factors, such as the nonlinear synergy between different types of demand^14^, and simultaneously generate the multiple energy predictions (including probabilistic ones^15^), where the data source is leveraged through support vector regression^16^, random forecast^17^, convolutional neural networks^18^, recurrent neural networks^18^, graph neural networks^19^, Transformer sequence-to-sequence networks^15,20^, and multi-task learning strategy^21^. Existing studies also assess the metabolic sustainability and the multiple energy interaction of universities as small-scale urban energy systems, where the data source is used for case studies^22,23^. In addition, a multi-objective optimization model that considers the interests of interconnected end-users in peer-to-peer energy trades is tested using the data source^24^. Numerous works demonstrate the wide range of research interests in multiple energy management. As current works mainly focus on the data source of multiple energy consumption of the entire region or several buildings, we contend that the scope for research and practical applications will not be fully realized unless we integrate comprehensive data sources, including meteorological information, PV power generation, and GHG emissions, to construct a complete, multi-source, and hierarchical dataset.

## Methods

### Raw data collection

The constructed dataset, HEEW, contains 11,987,328 records, involving diverse data sources. On the one hand, energy and GHG records derive from campus buildings of Arizona State University through the Campus Metabolism Project (http://cm.asu.edu/), a sensor-based system. These sensors are installed within the utility rooms of the buildings, in close proximity to the existing utility meters and circuit breakers, facilitating the immediate capture and logging of energy data. The accumulated data is then relayed to a central server machine, organized, and stored within a Structured Query Language (SQL) database. The server infrastructure is anchored by the Energy Information System, a system deployed in 2004 and maintained by the Arizona State University Facilities Management with aid from Ameresco. Upon being stored within the SQL database, a web-based tool can retrieve the data based on the user-defined viewing parameters and display it on-screen. It is worth noting that the sensor data is timestamped and reported to the central server, with data kept in onboard memory as a buffer to mitigate potential outages. In line with the project’s stated goal to support research and education, the original data was made publicly available by the Campus Metabolism, and has been widely used in the literature^14–24^. However, the original release had several limitations, including data gaps, minimal documentation, and a lack of integration with relevant environmental variables such as meteorological data. Specifically, 795,852 missing points occur in the data source of energy consumption, 12,625 in PV power generation, and 50364 in GHG emissions, which may affect the quality of raw data sources derived from the Campus Metabolism Project. The data for this study were downloaded on 23 September 2023. At the time of this writing, the project’s original website was inaccessible, and its availability has been noted to be intermittent. Since the real-world missing values have no ground truth, we develop a two-stage data cleaning scheme, as detailed in the following sections, which performs imputation and correction to generate artificial ground truth. The missing patterns structured by the proposed scheme can serve as a baseline for further investigations.

On the other hand, weather information is collected through the National Weather Service (https://www.weather.gov/), an agency of the United States federal government responsible for delivering weather-related products to organizations and the public. The equipment at the station records and reports a range of meteorological parameters, including temperature, humidity, wind speed, wind direction, atmospheric pressure, and precipitation. Similarly, there are 8356 missing points in the raw data source provided by the National Weather Service. Despite the inevitable occurrence of missing points in practical conditions, we believe in the application potential of integrating weather information into the multiple energy dataset. Hence, this study curates a comprehensive and user-ready version of the dataset and constructs the HEEW dataset. More specifically, we (1) conduct missing data imputation and abnormal data correction for missing or inconsistent records; (2) integrate publicly accessible meteorological records from the U.S. National Weather Service and other relevant data sources; and (3) apply standardized quality control procedures and harmonizing metadata for broader usability. All data processing, integration, and validation steps in this study have been completed using institutional computing resources and infrastructure.

### Buildings and end-use types

**Fig. 1:**
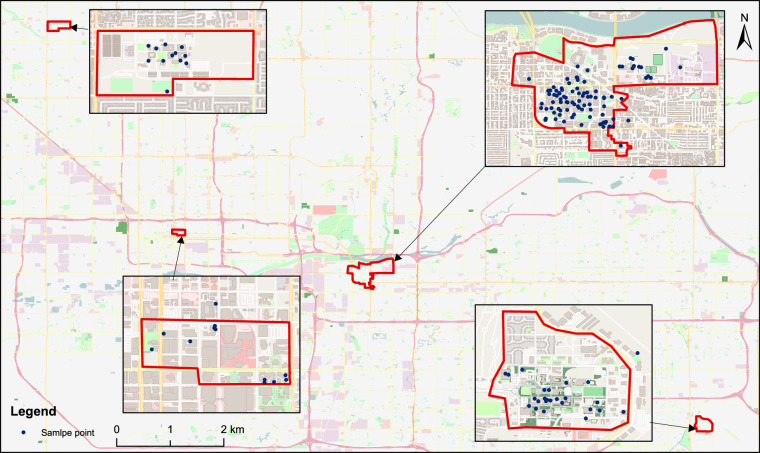
The distribution of individual buildings in four communities.

The data sources derive from the observations of sensors and meters installed in 147 individual buildings, four aggregated communities, and the entire region of Arizona State University. Figure 1 shows the spatial distribution of 147 individual buildings scattered in four communities of the entire region of Arizona State University. The individual buildings involve academic buildings (e.g., lecture halls, classrooms, and seminar rooms), research facilities (laboratories, research centers, and institutes), administrative offices (executive, financial, and administrative), student residences (dormitories, apartments, and residential colleges), recreational centers (fitness gyms, swimming pools, and sports arenas), performing arts venues (theaters, concert halls, and dance studios), libraries (scholarly resources, digital archives, and study spaces), dining halls and cafeterias (nutritional services and social spaces), the student union (a hub for student governance, organizations, and social interaction), health services (medical facilities, counseling centers, and wellness programs), conference centers (professional gatherings, symposia, and academic conferences), visitor accommodations (guest houses or hotels for visiting scholars, parents, and prospective students), and auxiliary services (bookstores, retail outlets, and other commercial services). The records cover all of the buildings in the Downtown Campus, the Polytechnic Campus, the Tempe Campus, and the West Valley Campus. These campuses in the entire region of Arizona State University are located in diverse areas and present varying patterns of energy demand. For instance, the Tempe Campus, located in Phoenix, Arizona, has a large demand for cooling energy because Phoenix, known for its torrid desert, has the highest average annual temperature in America. Overall, the end-use types include air conditioning, refrigeration, cooking, experimental processes or equipment, hot water, lighting, office equipment, and many other types. Both the chilled water and heating consumed in buildings are produced by either central plants or by the electricity that is already metered within a building.

### First-stage missing data imputation

Missing data, or the absence of data values in a dataset, refers to information that is not recorded or lost in practical conditions. The missing points can arise from a multitude of factors that are often contextually dependent on data acquisition from sensors or smart meters (e.g., sensor malfunctions, manual reporting errors, and environmental interferences), transmission via communication networks (communication failures, cybersecurity breaches, and protocol incompatibilities), storage in databases or data warehouses (database integrity issues, data archival practices, and access control errors), and even system-wide considerations (data integration complexities and resource constraints). To ensure the feasibility of the constructed dataset, HEEW, we develop a two-stage data cleaning scheme, which serves as a baseline to compare, as both the original raw data and the cleaned data processed by this scheme are made available to encourage comparative studies.

In the first stage, an empirical Threshold-Based Contextual Cleaning Algorithm (TCCA) is constructed as follows, considering the time-series characteristic of continuity and periodicity. Without loss of generality, the ratio *μ* is set as 5 to obtain the volatile threshold *θ* based on the upper quartile (UQ) of each column of the data sources (in the form of vectors *x*), thus deleting the abnormal values as Not a Number (NaN). Let AVERAGE be the function that returns the arithmetic mean of two input values, and RANDOM be the function that returns a random value within a specified range, where the left input denotes the lower bound and the right input denotes the upper bound. In instances where the absence of data is attributed to the original lack of sensor deployment, for instance, scenarios where PV panels have not been installed, such missing data is considered negligible. In other words, if the missing points occur in the whole column or a numeric value occurs from the first *i* + 1 rows, we assume these missing points are set to −1 for no installment situations. Importantly, energy data (including load and PV generation), GHG emissions, and weather conditions (such as temperature and humidity) are assumed to exhibit both temporal continuity, which means values change gradually over time, and periodicity, which reflects regular patterns over daily, weekly, or seasonal cycles^25^. Specifically, we expand linear interpolation into multiple correlation conditions^26–28^ and supplement each missing point by simultaneously searching the values from the last and following single timestamp and periods (e.g., day, week, and month), respectively, thus averaging both values that are not NaN values. For insurance, a random number within the interval between the minimum and maximum values of the column will be assigned to the missing value if both values that are not NaN values can not be found. Then, initial assignments from the first-stage missing data imputation will be improved based on the second-stage abnormal data correction.

#### Algorithm 1

Threshold-Based Contextual Cleaning (TCCA) Algorithm.

### Second-stage abnormal data correction

The noise is inevitably introduced to the actual observations during the processes of measurement, transmission, and storage, thereby impairing the quality of the data source. Specifically, noise manifests as minor spikes within raw data due to human-induced errors (such as recording by hand), discrepancies due to differing statistical methods, and the presence of outlier data. Human errors are particularly prevalent in sensors or smart meters that operate at high collection frequencies, have extended operational durations, or have lower levels of aggregation. Discrepancies in statistical methods may arise from differences in the types, manufacturers, and production standards of electrical equipment, sensors, or smart meters. In other words, human-induced errors and methodological discrepancies contribute to a portion of the data noise observed. Outlier data refers to statistical data affected by sudden events or specific causes, which can introduce significant random disturbances into standard time series, thereby influencing the accuracy of the values. If raw data is directly used as input for a model without any correction, the model’s output is assumed to deviate from the true values severely.

Hence, a Clustering-Based Ratio Algorithm for Correction (CRAC) is constructed as follows to double-check the abnormal values and improve the quality of the constructed dataset, HEEW. Let MIN be the function that returns the smaller value of two input numbers. The hourly resolution time series (e.g., electricity consumption) of each column is divided into daily curves. On the one hand, this study employs K-means clustering to extract typical daily curves and categorize all daily curves. Specifically, after pre-experiments, without loss of generality, the number of clusters is set at three, i.e., ${{\boldsymbol{c}}}_{1},{{\boldsymbol{c}}}_{2},{{\boldsymbol{c}}}_{3}$. Three days are selected from all the daily curves as the initial clustering centers by empirical pre-trials. The Euclidean distance ${\rm{DISTANCE}}\left({\boldsymbol{x}},{{\bf{X}}}_{{c}_{i}}\right)$ between a curve ***x*** and the typical curve ${{\bf{X}}}_{{c}_{i}}$ is calculated as follows, according to which the curve is put into the nearest cluster with the minimum distance ${\rm{MIN}}\left({\boldsymbol{d}}\right)$.

1 $${\rm{DISTANCE}}\left({\boldsymbol{x}},{{\bf{X}}}_{{c}_{i}}\right)=\sqrt{\mathop{\sum }\limits_{t=1}^{{\rm{LEN}}\left({\boldsymbol{x}}\right)}{\left({x}_{t}-{{\rm{X}}}_{{c}_{i},t}\right)}^{2}}$$

After each epoch, the typical curve is recalculated for each new cluster, which is repeated until the typical curves of all clusters remain unchanged. On the other hand, we assume the data points at the same moment in each cluster fluctuate in a certain (e.g., nearby) region composed of all the curves in the cluster. Thus, the abnormal values can be identified by setting the iteration ratio *ε*, which in this study is set to 3000, regarding the discrepancy between a single point ${{\boldsymbol{x}}}_{j}$ and the mean of all the points of the curves at the same moment, i.e., $\left({\sum }_{t}^{{\rm{LEN}}\left({\bf{Y}}\right)}{{\bf{Y}}}_{t,j}\right)/{\rm{LEN}}\left({\bf{Y}}\right)$. For variations exceeding the threshold ratio, the data points are assumed and identified as abnormal values, which will be corrected using the ratio method, considering the similarity assumption between adjacent time points as follows:

2 $${x}_{j}={y}_{j}\times \left(\frac{{x}_{j-1}}{{y}_{j-1}}+\frac{{x}_{j+1}}{{y}_{j+1}}\right)/2,i=p,\cdots ,q$$

In Eq. (2), the correction of the abnormal data can be realized by utilizing the normal data from the baseline central curve within the same cluster^29^. By exploiting the local similarity of the curves^30^, values from the corresponding position on the baseline curve within the same cluster can be transposed onto the curve in need of correction using the ratio method, thereby correcting the abnormal data. Hence, the abnormal data within initial assignments from the first-stage missing data imputation have been identified and corrected while avoiding the improper alteration of normal data. The improved assignments from the second-stage abnormal data correction ensure the quality of the dataset, HEEW.

#### Algorithm 2

 Clustering-based Ratio Algorithm for Correction (CRAC).

### Refined data estimation

GHG emissions are not directly collected in the constructed HEEW dataset. Instead, they are estimated as the difference between the emissions caused by the demand-side total energy consumption in each building and the emissions offset by solar energy production, which is evenly distributed across all metered buildings based on the total solar generation in the region. Specifically, the total energy consumption in mmBTU is calculated as the sum of electricity, cooling, and heat energy consumption. However, since these loads are expressed in different units, electricity in kilowatts (kW), cooling in refrigeration tons (Ton), and heat directly in mmBTU, they cannot be added directly. To ensure consistency, the electrical and cooling loads must first be converted to mmBTU/hr. For electricity, the conversion follows: kW to BTU/hr (using 1 kW ≈ 3,412 BTU/hr), then BTU/hr to mmBTU/hr (by dividing by 1,000,000). For cooling load, the process is: Ton to kW (1 Ton ≈ 3.51685 kW), then kW to BTU/hr to mmBTU/hr as above. The heating load, originally given in mmBTU, is interpreted as mmBTU/hr by assuming a 1-hour time resolution. Once all loads are expressed as mmBTU/hr, they can be summed to obtain the total energy consumption in a consistent unit.

Then, a distribution percentage is determined by calculating a building’s consumption against the total energy consumption and then using this same percentage against the total solar production value. The resulting amount of GHG is calculated by applying weighted emission factors to the building’s electric, chilled water, and heating loads separately and subtracting that allocation from the building based on the distribution percentage. In HEEW, the value of GHG is summed as follows based on the 2009 eGrid emission factors and corresponding multipliers. Since the energy consumed is initially measured in mmBTU, it is first converted to megawatt-hours (MWh) to facilitate the calculation of GHG emissions. Specifically, for every megawatt-hour (MWh) of energy consumed, the associated emissions are: 1191.35 lbs of carbon dioxide (CO₂), 0.01913 lbs of methane (CH₄), and 0.01558 lbs of nitrous oxide (N₂O). These are then converted to metric tons using a conversion factor of 2204.62 lbs per metric ton. The CH₄ and N₂O emissions are further multiplied by their respective global warming potential (GWP) factors, 21 for CH₄ and 310 for N₂O, to obtain their CO₂-equivalent impact.

## Data Records

### Data sources and content

The dataset is available at *figshare*^31^, and involves 147 individual buildings of Arizona State University in terms of multiple energy consumption, PV power generation, GHG emissions, and weather information. However, the record can be obtained after a certain time because the equipment was not installed in the building before, as mentioned in the section on Missing Data Imputation. Meanwhile, not all the dimensions can be provided for each building, as some equipment is not installed in the building. Table 1 summarizes the data sources available in HEEW.

**Table 1:** Summary of data sources in HEEW.

	EC	PV	GE	W		EC	PV	GE	W		EC	PV	GE	W
*Total*	ALL	√	√	√	BN048	E		√		BN099	E	√	√	
*CN001*	ALL	√	√		BN049	E		√		BN100	ALL	√	√	
BN001	ALL		√		BN050	E	√	√		BN101	ALL		√	
BN002	E&C	√	√		BN051	ALL		√		BN102	ALL		√	
BN003	E		√		BN052	ALL	√	√		BN103	ALL		√	
BN004	E		√		BN053	E		√		BN104	ALL		√	
BN005	E		√		BN054	ALL	√	√		BN105	E&H	√	√	
BN006	E		√		BN055	E&H	√	√		BN106	ALL		√	
BN007	E		√		BN056	ALL	√	√		BN107	E&C		√	
BN008	E		√		BN057	ALL	√	√		BN108	E&C		√	
BN009	E		√		BN058	ALL		√		BN109	E&C		√	
BN010	E&C		√		BN059	ALL		√		BN110	E&H		√	
BN011	E		√		BN060	ALL		√		BN111	E&C		√	
CN002	ALL	√	√		BN061	ALL		√		BN112	E&C		√	
BN012	E		√		BN062	ALL		√		BN113	E&C		√	
BN013	E		√		BN063	ALL	√	√		BN114	ALL	√	√	
BN014	E&C		√		BN064	E	√	√		BN115	ALL	√	√	
BN015	E		√		BN065	E&C		√		BN116	ALL		√	
BN016	E		√		BN066	E	√	√		BN117	E		√	
BN017	E&C		√		BN067	ALL		√		BN118	ALL		√	
BN018	E&C		√		BN068	E		√		BN119	E&C	√	√	
BN019	E		√		BN069	ALL	√	√		BN120	ALL		√	
BN020	ALL		√		BN070	ALL	√	√		BN121	E		√	
BN021	E&C		√		BN071	ALL	√	√		BN122	E&C		√	
BN022	E		√		BN072	E&C	√	√		BN123	E&C		√	
BN023	E		√		BN073	E&C		√		BN124	E&C		√	
BN024	E		√		BN074	E&C	√	√		BN125	E		√	
BN025	E		√		BN075		√	√		BN126	E	√	√	
BN026	E&C		√		BN076	ALL	√	√		BN127	E	√	√	
BN027	E		√		BN077	E		√		BN128	E&C		√	
BN028	E&C		√		BN078	ALL		√		BN129	E	√	√	
BN029	E&C		√		BN079	ALL	√	√		BN130	E	√	√	
BN030	E		√		BN080	ALL		√		BN131	E	√	√	
BN031	E		√		BN081	ALL		√		BN132	ALL	√	√	
BN032	E&C	√	√		BN082	E	√	√		BN133	ALL		√	
BN033	E&C		√		BN083	ALL		√		BN134	E		√	
BN034	E&C		√		BN084	ALL		√		BN135	E		√	
BN035	E		√		BN085	ALL		√		BN136	E&C	√	√	
BN036	E&C		√		BN086	ALL		√		CN004	E&C	√	√	
CN003	ALL		√		BN087	E&H		√		BN137	E&C		√	
BN037	E	√	√		BN088	ALL		√		BN138	E&C		√	
BN038	ALL		√		BN089	E&C	√	√		BN139	E		√	
BN039	E		√		BN090	ALL		√		BN140	E&C		√	
BN040	ALL	√	√		BN091	E	√	√		BN141	E&C		√	
BN041	ALL	√	√		BN092	ALL		√		BN142	E&C		√	
BN042	ALL	√	√		BN093	E&C		√		BN143	E&C		√	
BN043	ALL		√		BN094	ALL	√	√		BN144	E&C	√	√	
BN044	E&C		√		BN095	ALL		√		BN145	E&C		√	
BN045	ALL	√	√		BN096	ALL		√		BN146	E&C		√	
BN046	E&C		√		BN097	E&C	√	√		BN147	E&C		√	
BN047	ALL		√		BN098	E	√	√						

CN: Community No., Building No., EC: Energy Consumption, E: Electricity [kW], H: Heat [mmBTU], C: Cooling Energy [Ton], PV: PV Power Generation [kW], GE: Greenhouse Gas Emission [Ton], W: Weather, including Temperature [°F], Dew Point [°F], Humidity [%], Wind Speed [mph], Wind Gust [mph], Sea Level Pressure [in], Precipitation [in].

In Table 1, all electric loads in buildings come either from PV output or the utility grid. It is also shown that not all buildings are equipped with the district heating system and PV panels, and some buildings are only equipped with electric load or electric and cooling loads. The building dubbed BN075 is only installed with PV panels without any loads, and weather information is only available for area-level aggregated communities. In addition, net GHG emissions are calculated considering the gap between emission increase due to energy consumption and emission reduction due to PV power generation.

### Data record glossary

Table 2 summarizes the data record glossary in the constructed dataset, HEEW. The calendar information involves year, month, day, hour, and weekday. The timestamp is composed of year, month, day, and hour, in which the year ranges from 2014 to 2022, and the month refers to January, February, March, April, May, June, July, August, September, October, November, and December. The weekday refers to Monday, Tuesday, Wednesday, Thursday, Friday, Saturday, and Sunday.

**Table 2:** Glossary of data records in HEEW.

Column header	Explanation and unit
Year, Month, Day, Hour, Weekday	Year, month, day, hour, and weekday are calendar information. The timestamp is composed of year, month, day, and hour, in which the year ranges from 2014 to 2022.
Electricity	Electricity is the electrical energy used to energize lighting systems, electronic devices, and ventilation equipment. The unit used to denote the rate of electricity consumption is the kilowatt (kW).
PV	PV power generation is the electrical energy produced to supply electricity consumption. The unit used to denote the rate of PV power generation is also kW, which signifies the instantaneous electrical power output provided by the solar installation.
Cooling	Cooling energy is the energy used to cool the water and air within buildings. The unit used to denote the rate of cooling energy consumption is the Ton.
Heat	Heat energy is the energy used to heat the water and air in the building. The unit used to quantify heat energy consumption is the British Thermal Unit (Btu), which in HEEW is expressed in the form of mmBtu (1,000,000 Btu).
Total Energy	The total energy is the sum of electricity consumption given in the form of mmBtu (1 kWh = 3.4 mBtu), cooling energy consumption in the form of mmBtu (1 Tonh = 11.972 mBtu), and heat energy consumption.
Emission	GHG emission is calculated as the gap between emission increase due to energy consumption and emission reduction due to PV power generation. The unit used to denote the GHG emission is the Ton.
Temperature	Temperature is a measure of the thermal energy present in a substance or object. The unit used to denote the measurement of temperature is the degree Fahrenheit (°F), which is a scale on which water freezes at 32 degrees and boils at 212 degrees under standard atmospheric conditions.
Dew Point	The dew point is the temperature at which air becomes saturated with moisture, and water vapor begins to condense into dew. The unit used to denote the measurement of the dew point is also the degree Fahrenheit (°F).
Humidity	Humidity is the amount of water vapor present in the air. The unit used to denote the measurement of humidity is the percentage (%) to quantify the degree to which the air is saturated with moisture.
Wind Speed	Wind speed is the velocity at which air moves from areas of high pressure to areas of low pressure. The unit used to measure wind speed is miles per hour (mph), which indicates the number of miles the wind travels in one hour.
Wind Gust	The wind gust is a brief and sudden increase in wind speed that typically lasts for just a few seconds. The unit used to measure the wind gust is also miles per hour (mph), conveying the distance, in miles, that the gust travels within one hour.
Sea Level Pressure	Sea Level Pressure is a measure of the atmospheric pressure at sea level. The unit used to measure sea level pressure is inches of mercury (in). The standard sea level pressure is defined as 29.92 inches of mercury under standard gravitational conditions at a temperature of 32 degrees Fahrenheit (0 degrees Celsius).
Precipitation	Precipitation is various forms of moisture, such as rain, snow, sleet, and hail, that falls from clouds and reaches the ground. The unit used to denote the measurement of precipitation is inch (in).

Electricity is the electrical energy used to energize lighting systems, electronic devices, and ventilation equipment. The quantification of electricity consumption is gauged by a power meter, which is installed at the electrical ingress of the building.

Cooling energy is the energy used to cool the water and air within buildings. At Arizona State University, the central utility facility cools the water through electric chillers, and the cool water is then distributed to various buildings. The quantification of cooling energy consumption is calculated through the thermal increment and volumetric flow rate of the cool water that circulates into and out of the facility [14].

Heat energy is the energy used to heat the water and air in the building. Arizona State University is equipped with a central plant where natural gas-fired burners are employed to heat water, which is thereafter channeled to the various buildings across the campus [14]. The heat consumed by buildings primarily originates from such centralized sources, and heat pumps may also contribute to heating in some cases, depending on the mechanical system design and energy infrastructure. Moreover, the total energy consumption is also calculated as the sum of electricity, cooling energy, and heat energy consumption for generic purposes.

Greenhouse gas emissions result from greenhouse gases that contribute to the greenhouse effect by absorbing infrared radiation produced by solar warming of the Earth’s surface, including carbon dioxide (CO2), Methane (CH4), and nitrous oxide(NO2). Greenhouse gas emission is calculated as the gap between emission increase due to electricity consumption in each building and the emission reduction via solar production, equally distributed to each metered building.

PV power generation is the electrical energy produced to supply electricity consumption. At Arizona State University, PV panels are deployed atop certain buildings within the campus grounds. The capacity of these PV panels to generate electricity is also quantified in kW.

Weather information involves environmental factors, such as temperature, dew point, humidity, wind speed, wind gust, sea level pressure, and precipitation, provided by stations of the National Weather Service around the four communities. Specifically, temperature, a measure of the thermal energy present in a substance or object, serves as an indicator of the degree of heat or coldness as perceived by the sense of touch or determined by a thermometer.

The dew point is a precise measure of atmospheric moisture, indicating the temperature at which the air can no longer “hold” all of its water vapor, which then starts to condense into liquid water or dew, which is measured using a hygrometer or derived from other meteorological data through calculations involving temperature and relative humidity readings. A higher dew point signifies greater moisture content in the air, leading to a feeling of “mugginess” or “stickiness” as the moisture content approaches the saturation point. Conversely, a lower dew point means drier air and lower relative humidity, providing a more comfortable and less humid environment.

Humidity is commonly measured using instruments such as hygrometers, reflecting the current state of water vapor relative to the maximum amount that the air can hold at a given temperature, known as the saturation point. When the humidity level is expressed as 100%, the air is at full saturation, which often leads to the formation of dew, fog, or precipitation, as the air cannot hold any additional moisture. Conversely, lower humidity levels indicate drier air.

Wind speed is typically measured using an anemometer, a device specifically designed to gauge the speed of the wind with a high degree of accuracy. Wind gusts are short bursts of high-speed wind that are often associated with rapidly changing weather conditions, such as the approach of a storm front or convective activity within the atmosphere. To capture these transient events, anemometers, when measuring wind gusts, record the peak wind speed over a short interval, typically ranging from a few seconds to a minute, to ensure an accurate representation of the gust’s maximum force.

Sea level pressure indicates areas of high and low pressure, which is standardized to provide a reference that can be compared across various geographic locations, regardless of altitude, to ensure consistency in pressure readings. Barometric pressure sensors and mercury barometers are commonly used tools for measuring this atmospheric pressure parameter, with one inch of mercury representing the pressure exerted by a one-inch-high column of mercury at the Earth’s surface.

The amount of precipitation is recorded in terms of the depth of water that would accumulate on an impermeable, flat surface within a specified period. Precipitation is typically measured using a rain gauge, which collects falling water and allows for direct measurement of the depth, with an inch of precipitation meaning that a one-inch deep layer of water has fallen over a one-square-inch area.

To align with the Findable, Accessible, Interoperable, and Reusable (FAIR) data principles, we present a metadata summary of the 13 key variables, as shown in Table 3, including variable names, units, temporal resolution, data availability, and data sources. Based on Tables 1–4, researchers can quickly understand the structure of the HEEW dataset and efficiently leverage it for further investigation.

**Table 3:** Metadata summary.

Variable	Unit	Frequency	Availability	Source
Electricity	kW	Hourly	99.3% buildings and their aggregations in the entire region (2014–2022)	Meters/sensors
PV	kW	Hourly	29.9% buildings and their aggregations in the entire region (2014–2022)	Meters/sensors
Cooling	Ton	Hourly	66.0% buildings and their aggregations in the entire region (2014–2022)	Meters/sensors
Heat	mmBTU	Hourly	37.4% buildings and their aggregations in the entire region (2014–2022)	Meters/sensors
Total Energy	mmBTU	Hourly	99.3% buildings and their aggregations in the entire region (2014–2022)	Calculation
Emission	Ton	Hourly	Full (2014–2022)	Estimation
Temperature	°F	Hourly	The entire region (2014–2022)	Weather station
Dew Point	°F	Hourly	The entire region (2014–2022)	Weather station
Humidity	%	Hourly	The entire region (2014–2022)	Weather station
Wind Speed	mph	Hourly	The entire region (2014–2022)	Weather station
Wind Gust	mph	Hourly	The entire region (2014–2022)	Weather station
Sea Level Pressure	in	Hourly	The entire region (2014–2022)	Weather station
Precipitation	in	Hourly	The entire region (2014–2022)	Weather station

**Table 4:** Summary of ranges, missing rates, and abnormal rates of the HEEW data.

	Range	MR	AR		Range	MR	AR		Range	MR	AR
**Total**	2014~22	0	0	BN048	2015~22	0	0	BN099	2014~22	0	0
*CN001*	2014~22	0	0	BN049	2014~22	0	0	BN100	2014~22	0	0
BN001	2016~22	0	0	BN050	2014~22	0	0	BN101	2014~22	0	0
BN002	2014~22	0	0	BN051	2014~22	0	0	BN102	2014~22	0	0
BN003	2014~22	0	0	BN052	2014~22	0	0	BN103	2014~22	0	0
BN004	2014~22	0	0	BN053	2015~22	0	0	BN104	2014~22	0	0
BN005	2014~22	0	0	BN054	2014~22	0	0	BN105	2014~22	0	0
BN006	2014~22	0	0	BN055	2014~22	0	0	BN106	2014~22	0	0
BN007	2014~22	0	0	BN056	2014~22	0	0	BN107	2014~22	0	0
BN008	2014~22	0	0	BN057	2014~22	0	0	BN108	2014~22	0	0
BN009	2014~22	0	0	BN058	2014~22	0	0	BN109	2014~22	0	0
BN010	2014~22	0	0	BN059	2014~22	0	0	BN110	2014~22	0	0
BN011	2014~22	0	0	BN060	2014~22	0	0	BN111	2014~22	0	0
CN002	2014~22	0	0	BN061	2014~22	0	0	BN112	2014~22	0	0
BN012	2014~22	0	0	BN062	2014~22	0	0	BN113	2014~22	0	0
BN013	2014~22	0	0	BN063	2014~22	0	0	BN114	2014~22	0	0
BN014	2014~22	0	0	BN064	2014~22	0	0	BN115	2014~22	0	0
BN015	2014~22	0	0	BN065	2014~22	0	0	BN116	2014~22	0	0
BN016	2014~22	0	0	BN066	2014~22	0	0	BN117	2014~22	0	0
BN017	2014~22	0	0	BN067	2014~22	0	0	BN118	2014~22	0	0
BN018	2018~22	0	0	BN068	2019~22	0	0	BN119	2014~22	0	0
BN019	2014~22	0	0	BN069	2014~22	0	0	BN120	2014~22	0	0
BN020	2014~22	0	0	BN070	2014~22	0	0	BN121	2014~22	0	0
BN021	2014~22	0	0	BN071	2014~22	0	0	BN122	2017~22	0	0
BN022	2014~22	0	0	BN072	2014~22	0	0	BN123	2017~22	0	0
BN023	2014~22	0	0	BN073	2014~22	0	0	BN124	2017~22	0	0
BN024	2014~22	0	0	BN074	2014~22	0	0	BN125	2014~22	0	0
BN025	2014~22	0	0	BN075	2014~22	0	0	BN126	2014~22	0	0
BN026	2014~22	0	0	BN076	2014~22	0	0	BN127	2014~22	0	0
BN027	2014~22	0	0	BN077	2014~22	0	0	BN128	2014~22	0	0
BN028	2014~22	0	0	BN078	2014~22	0	0	BN129	2014~22	0	0
BN029	2014~22	0	0	BN079	2014~22	0	0	BN130	2014~22	0	0
BN030	2014~22	0	0	BN080	2014~22	0	0	BN131	2014~22	0	0
BN031	2014~22	0	0	BN081	2014~22	0	0	BN132	2014~22	0	0
BN032	2014~22	0	0	BN082	2014~22	0	0	BN133	2014~22	0	0
BN033	2014~22	0	0	BN083	2014~22	0	0	BN134	2014~22	0	0
BN034	2014~22	0	0	BN084	2014~22	0	0	BN135	2014~22	0	0
BN035	2014~22	0	0	BN085	2014~22	0	0	BN136	2014~22	0	0
BN036	2014~22	0	0	BN086	2014~22	0	0	CN004	2014~22	0	0
CN003	2014~22	0	0	BN087	2014~22	0	0	BN137	2014~22	0	0
BN037	2014~22	0	0	BN088	2014~22	0	0	BN138	2014~22	0	0
BN038	2014~22	0	0	BN089	2014~22	0	0	BN139	2014~22	0	0
BN039	2014~22	0	0	BN090	2014~22	0	0	BN140	2014~22	0	0
BN040	2014~22	0	0	BN091	2014~22	0	0	BN141	2014~22	0	0
BN041	2014~22	0	0	BN092	2014~22	0	0	BN142	2017~22	0	0
BN042	2014~22	0	0	BN093	2014~22	0	0	BN143	2014~22	0	0
BN043	2018~22	0	0	BN094	2014~22	0	0	BN144	2014~22	0	0
BN044	2014~22	0	0	BN095	2014~22	0	0	BN145	2014~22	0	0
BN045	2014~22	0	0	BN096	2014~22	0	0	BN146	2014~22	0	0
BN046	2014~22	0	0	BN097	2014~22	0	0	BN147	2014~22	0	0
BN047	2014~22	0	0	BN098	2014~22	0	0				

MR: Missing rate, AR: Abnormal rate [%].

### Hierarchical data record benchmarking

**Fig. 2:**
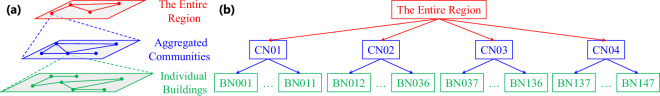
Hierarchical illustration of the HEEW data. (**a**) Hierarchical illustration from individual buildings to aggregated communities to the entire region. (**b**) Hierarchy of the HEEW data, where the numbers at the bottom level indicate the number of buildings.

The records of 147 buildings within the entire region aggregate to constitute four records of the four distinct communities, and the records of the four communities aggregate to generate the record of the entire region. This integrative process begets a comprehensive compilation of records, which, upon subsequent synthesis, culminates in the hierarchical dataset, HEEW. Figure 2 illustrates the three-level hierarchy of the HEEW data from individual buildings to four communities to the entire region, where the buildings belong to varying communities based on geographic locations and administrative boundaries, as shown in Fig. 1. In HEEW, the values in the record of each community are derived by the element-wise summation of values in the records of its buildings. For instance, the electricity consumption of the CN01 record on 15 September, 2022, is the sum of the electricity consumption of the records from BN001 to BN011 on 15 September, 2022.

### File organization

The HEEW dataset contains 152 files, each cataloging pertinent data on energy and climatic conditions. These files are systematically named and categorized based on the type of data they contain and the location from which the data were collected. The *Total_energy.csv* file encapsulates the aggregate energy consumption for the entire dataset, serving as a comprehensive summary of energy data across all buildings. Following this, there is a meteorological data file named *Total_weather.csv*, covering the entire region. These files furnish climate information relevant to energy consumption and production, such as temperature, humidity, and solar radiation, corresponding to various geographic regions or meteorological stations.

In addition, files named *CN01_energy.csv* through *CN04_energy.csv* contain energy consumption records of individual buildings within specific communities, with the numbers in the filenames designating different regions or building clusters. The prefix CN stands for Community Number, indicating the code for the respective area or cluster of buildings. The remaining files, prefixed with BN and enumerated from *BN001_energy.csv* to *BN147_energy.csv*, include the energy consumption data for individual buildings. In these filenames, BN denotes Building Number, with the subsequent three digits uniquely identifying each building. This systematic approach to file naming and organization enables clear identification of the content and source of each file, laying a solid foundation for in-depth analysis of energy consumption and weather data. Such an organizational structure also facilitates efficient data retrieval and processing by researchers according to their specific needs.

## Technical Validation

In this section, we validate the quality and feasibility of our constructed dataset, HEEW, by checking data gaps and consistency. Table 4 summarizes the timestamp ranges, missing rates, and abnormal rates of each multi-source data source in HEEW.

### Data gap check

In this part, the data gap, the degree of data completeness, is represented as the missing rate of the HEEW data, which can be obtained by checking the missing points. As the 152 data sources vary in the dynamic range of timestamps, the missing points have been set to 0 for the situations in which some equipment was not installed until a certain time or has never been installed in the past. Through the Threshold-Based Contextual Cleaning Algorithm (TCCA) for imputation, we have comprehensively addressed the issue of the missing entries, achieving a missing rate of zero percent within the constructed HEEW dataset. Thus, the data gap has been double-checked to guarantee the completeness of HEEW to a high level of confidence.

### Data consistency check

**Fig. 3:**
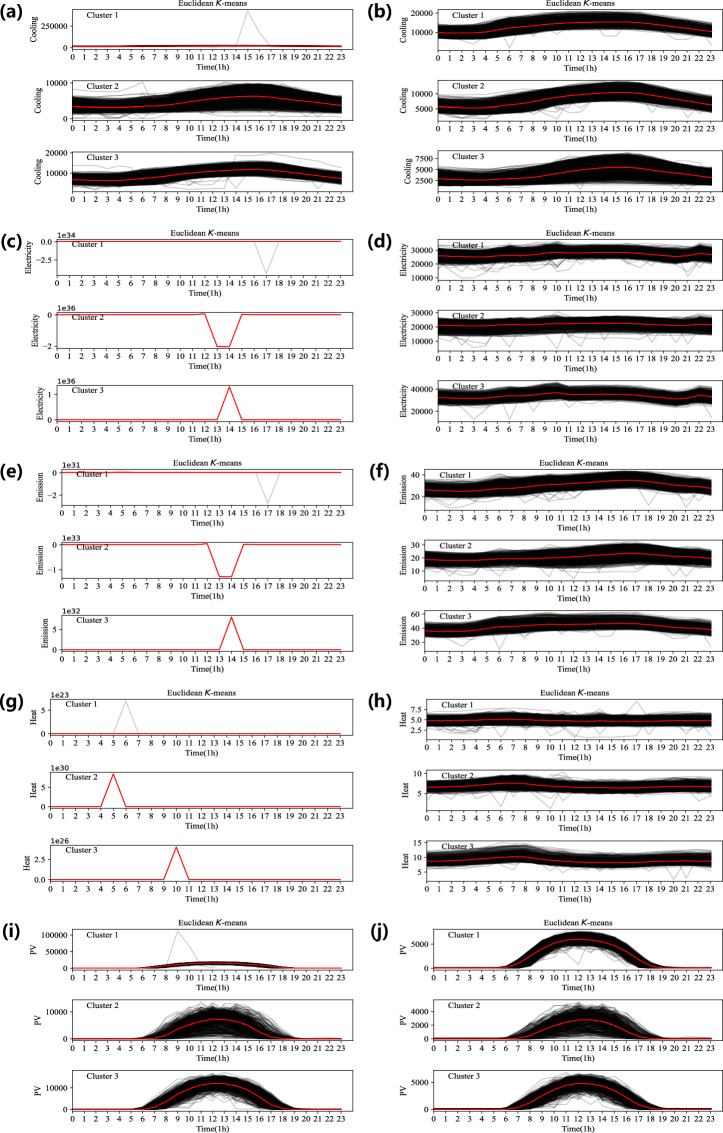
The K-means clustering results on the raw and cleaned data at the top level of the proposed dataset’s hierarchical structure. (**a**) PV output before correction. (**b**) PV output after correction. (**c**) Electric load before correction. (**d**) Electric load after correction. (**e**) Heat load before correction. (**f**) Heat load after correction. (**g**) Cooling load before correction. (**h**) Cooling load after correction. (**i**) Greenhouse gas emissions before correction. (**j**) Greenhouse gas emissions after correction.

**Fig. 4:**
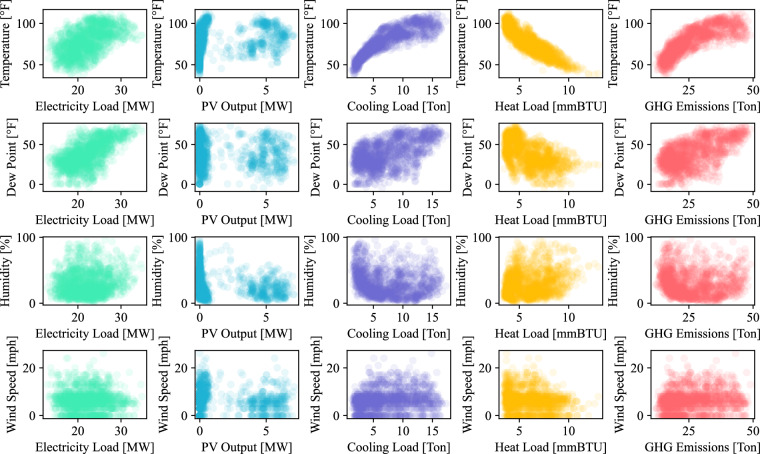
Correlation scatter plot between energy and weather records.

In this part, we, on the one hand, evaluate the internal consistency in terms of the time-series characteristic of data sources from Arizona State University and, on the other hand, validate the integration consistency of multiple data sources from Arizona State University and those from the National Weather Service. For internal consistency, the abnormal rate of multiple energy consumption, PV power generation, and GHG emissions in the HEEW data is obtained by testing the abnormal points. Using the TCCA and CRAC, we have comprehensively addressed the issue of the abnormal entries on the fully reconstructed dataset with missing values already imputed, validating the developed algorithms for the HEEW dataset. Figure 3 shows the K-Means clustering results of the raw and cleaned data (e.g., multiple energy consumption, PV power generation, and GHG emissions) at the top level within HEEW as an instance. For integration consistency, the correlation between energy records and weather records (temperature, dew point, humidity, and wind speed) of the entire region for 2014 is exhibited as an instance, as shown in Fig. 4.

**Fig. 5:**
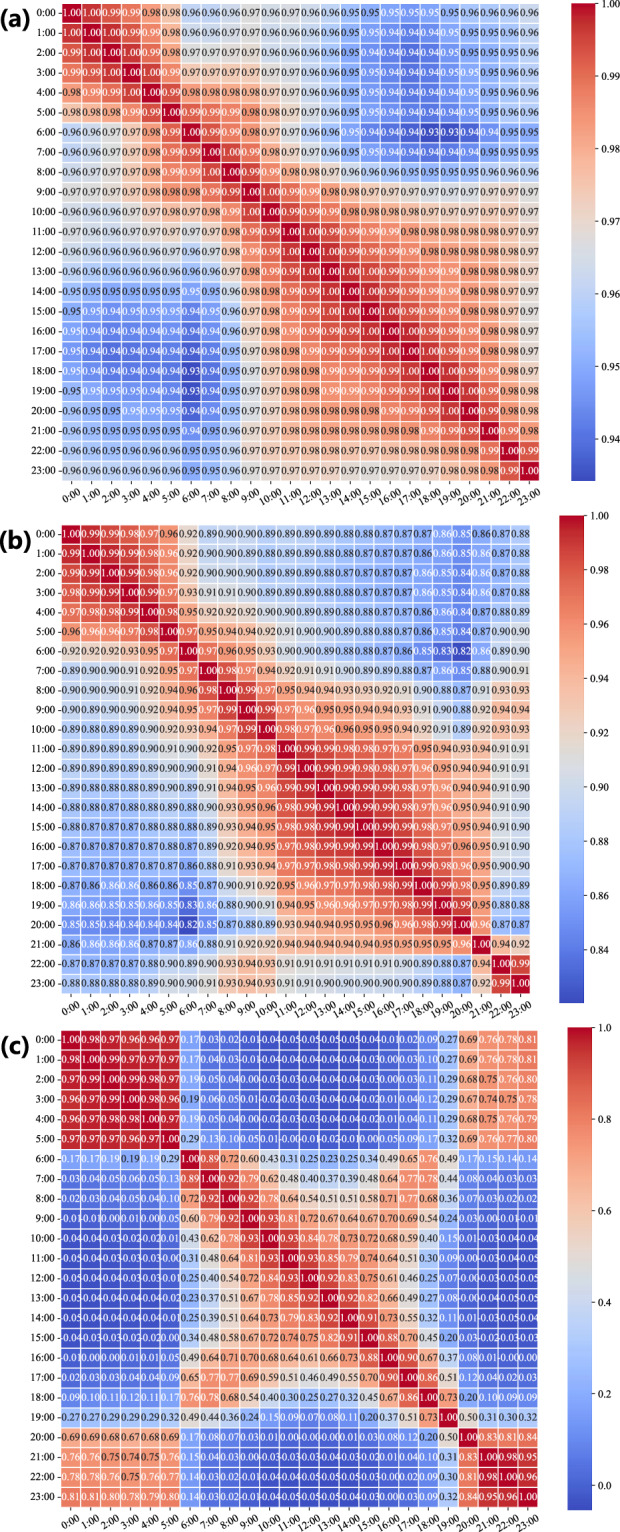
Correlation coefficient matrices from previous hours. (**a**) Electricity consumption. (**b**) PV power output. (**c**) Greenhouse gas emissions.

**Fig. 6:**
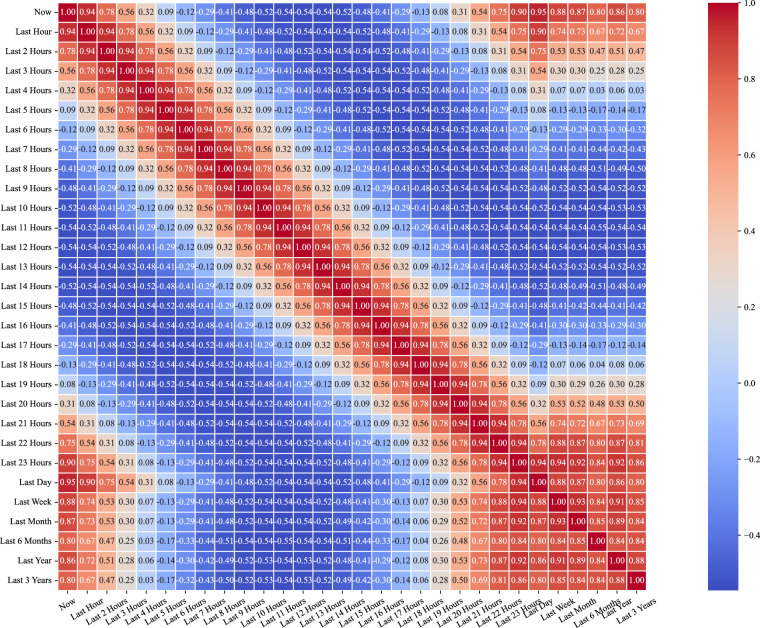
Correlation coefficient matrices of PV output from the past year.

**Fig. 7:**
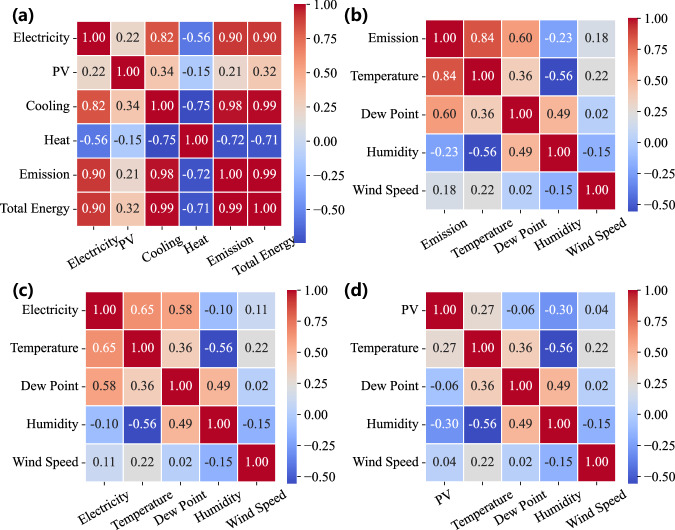
Correlation coefficient matrices between various variables. (**a**) Energy consumption, PV output, and emissions. (**b**) Electricity consumption and weather. (**c**) PV power output and weather. (**d**) GHG emissions and weather.

**Fig. 8:**
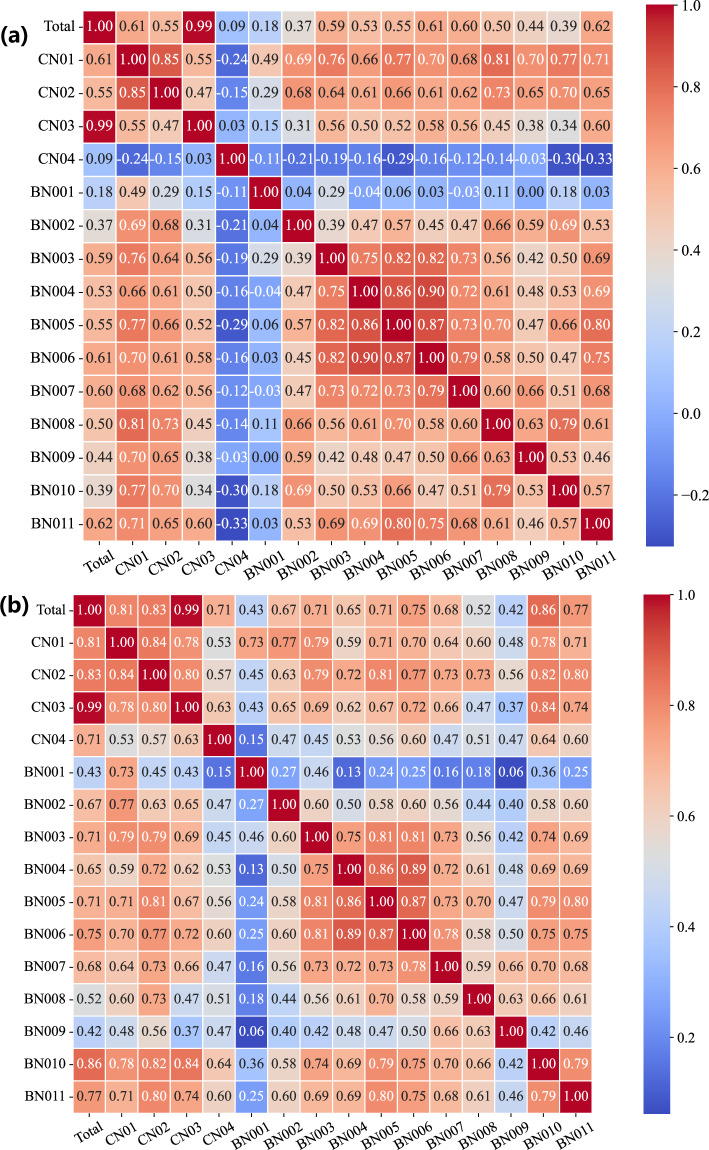
Correlation coefficient matrices of the hierarchical components. (**a**) Electricity consumption. (**b**) GHG emissions.

Furthermore, we test the temporal-spatial summation consistency of multiple data sources from aggregated communities and those from individual buildings. Firstly, for each entry in the community records, we meticulously matched and reviewed the corresponding records of all buildings at the same time point. Taking the record of CN03 on 15 September, 2021, as an example, we checked the data of all buildings from BN037 to BN136 on that day. By summing up the records of these buildings, we verify that the recorded total for the community matches the sum of the records from buildings, and the discrepancy points are then obtained. Then, as programmatic scripts are developed to automate the aggregation process, we examine each community record to ensure that each is indeed the sum of the records of its constituent buildings. In addition, to rule out potential errors or discrepancies during the aggregation process, we implement multiple rounds of data cross-checking mechanisms. If any deviation between the aggregated data and the sum of the building data is detected, we reorganize and recalculate the data until the records of all communities are consistent. Through this data verification process, we can confidently assert that every community record in the HEEW dataset precisely represents the sum of the records from the buildings within it. Figures 5–8 show three correlation coefficient matrices of electricity consumption, PV power output, and GHG emissions, respectively, from varying aspects. Thus, the data consistency has been double-checked to guarantee the feasibility and quality of HEEW to a high level of confidence.

## Data Availability

The dataset is available at figshare (10.6084/m9.figshare.28425647).

## Code availability

The code used for constructing and processing the HEEW dataset described in this study is accessible in the GitHub repository at (https://github.com/hanjiang98/HEEW-dataset.git). Throughout the study, we mainly employ the Windows 11 operating system, with Python 3.11 as the programming language. For data manipulation, the pandas library version 2.1.4 and numpy version 1.24.3 are used, while matplotlib 3.7.1 serves for data visualization purposes, and the time series learning library tslearn is adopted at version 0.6.2^32^. Specific parameters are set for the generation, testing, or processing of the current dataset, as mentioned in the Methods section. Without loss of generality, the threshold ratio *μ* is empirically determined through preliminary experiments and is set to 5. The number of clusters is set to three, based on both pre-experimental results and references from related literature^25^. The iteration ratio ε is configured to 3000, also informed by preliminary trials to ensure convergence and computational efficiency. The random seed is fixed as 77 for synthesizing missing values. The settings of these parameters can be flexible through trials and pre-experiments.
